# Morphological Heterogeneity of Large (Koos Grade 4) Vestibular Schwannomas: Surgical Consequences and Predictors of Facial Nerve Outcome

**DOI:** 10.3390/jcm15187153

**Published:** 2026-09-15

**Authors:** Julia Shawarba, Marlene Urbas, Christoph Arnoldner, Christian Matula, Karl Roessler

**Affiliations:** 1 Department of Neurosurgery, Medical University of Vienna, 1090 Vienna, Austria; julia.shawarba@meduniwien.ac.at (J.S.); marlene.urbas@meduniwien.ac.at (M.U.); christian.matula@meduniwien.ac.at (C.M.); 2 Comprehensive Center of Clinical Neurosciences and Mental Health (C3N-MH), Medical University of Vienna, 1090 Vienna, Austria; 3 Department of Otorhinolaryngology, Medical University of Vienna, 1090 Vienna, Austria; christoph.arnoldner@meduniwien.ac.at

**Keywords:** vestibular schwannoma, Koos grade 4, facial nerve, microsurgery, brainstem compression, outcome prediction

## Abstract

**Background/Objectives:** Microsurgery is the principal treatment for large vestibular schwannomas (VS) causing symptomatic brainstem compression. Koos grade 4 tumors remain anatomically heterogeneous. We evaluated surgical and functional outcomes and explored whether a study-specific subdivision, adapted from the Hannover T4a/T4b distinction, provides prognostic information beyond established clinical and surgical variables. **Methods:** This retrospective single-center study included 139 adults treated for unilateral Koos grade 4 VS between January 2013 and December 2024. Tumors were categorized as Koos 4a (brainstem compression without fourth ventricular compression) or Koos 4b (brainstem displacement with fourth ventricular compression). The primary endpoint was facial nerve function at the last documented clinical follow-up. Resection extent was determined by preoperative and early postoperative contrast-enhanced MRI. **Results:** Seventy-five patients (54.0%) had Koos 4a and 64 (46.0%) Koos 4b tumors. Koos 4b tumors were larger (median 38 vs. 30 mm; *p* < 0.001). Gross-total resection was achieved in 73.5%, and complications occurred in 13.7%. Favorable facial nerve function (House–Brackmann grades I–II) decreased from 98.5% preoperatively to 36.0% immediately postoperatively and was documented in 58.7% at the last available follow-up. In exploratory complete-case multivariable analysis (n = 104), longer operative duration was associated with lower odds of favorable facial nerve function (adjusted OR per 30 min, 0.84; 95% CI, 0.74–0.95; *p* = 0.005). This association should not be interpreted causally because operative duration likely reflects tumor complexity. The study-specific subtype was not associated with outcome after adjustment, but collinearity with tumor diameter and limited model precision preclude a definitive conclusion. **Conclusions:** This study-specific subdivision describes differences in anatomical extent and preoperative clinical burden. Its independent prognostic value was not demonstrated in this single-center cohort and requires external validation. Operative duration appears to be a marker of surgical complexity rather than an actionable causal risk factor.

## 1. Introduction

Vestibular schwannomas (VS) are benign World Health Organization grade I neoplasms arising from Schwann cells of the vestibular division of the eighth cranial nerve. They account for approximately 80–90% of cerebellopontine angle tumors and represent one of the most common pathologies encountered in skull base surgery. Although their biological behavior is generally indolent, progressive enlargement may result in compression of the brainstem, cerebellum, and adjacent cranial nerves, leading to hearing loss, facial nerve dysfunction, tinnitus, trigeminal neuropathy, gait disturbance, hydrocephalus, and ultimately life-threatening brainstem compression [1,2].

Treatment strategies for vestibular schwannomas include observation, stereotactic radiosurgery, and microsurgical resection. While conservative management and radiosurgery are established options for small- and medium-sized tumors, microsurgical decompression remains the principal treatment for large tumors with symptomatic mass effect or brainstem compression. Contemporary skull base surgery increasingly emphasizes functional preservation and durable tumor control rather than unconditional gross-total resection [2,3,4,5]. The Koos classification is widely used to stratify vestibular schwannomas according to tumor size and relationship to the brainstem; grade 4 denotes brainstem displacement. The Hannover system offers a more granular description of advanced tumors: T4a lesions compress the brainstem, whereas T4b lesions additionally produce severe brainstem deformation and fourth ventricular compression or displacement [5,6,7]. Previous Hannover T4a/T4b series indicate that fourth ventricular involvement marks greater anatomical and preoperative neurological burden, but they have not established that this feature provides independent prognostic information for facial nerve recovery beyond tumor size and operative complexity [7,8,9]. The present study therefore uses a study-specific Koos 4a/4b terminology, explicitly adapted from rather than proposed as a replacement for the Hannover classification, to examine this unresolved question within a cohort uniformly meeting Koos grade 4 criteria. We evaluated radiological, surgical, and functional outcomes and explored whether this subdivision was associated with perioperative morbidity, extent of resection, and facial nerve function at the last documented follow-up.

## 2. Materials and Methods

### 2.1. Study Design and Patient Selection

This retrospective single-center cohort study included all consecutive adult patients who underwent surgical treatment for a unilateral Koos grade 4 vestibular schwannoma at the Department of Neurosurgery, Medical University of Vienna, between 1 January 2013 and 31 December 2024. This study was approved by the Ethics Committee of the Medical University of Vienna prior to data collection (2365/2025 Medical University Vienna), and all patient data were anonymized in accordance with institutional regulations and the Declaration of Helsinki. The requirement for study-specific informed consent was waived by the Ethics Committee due to the retrospective nature of this study. Additionally, all patients had provided written consent before surgery permitting the anonymized use of their clinical data for future research.

**Figure 1:**
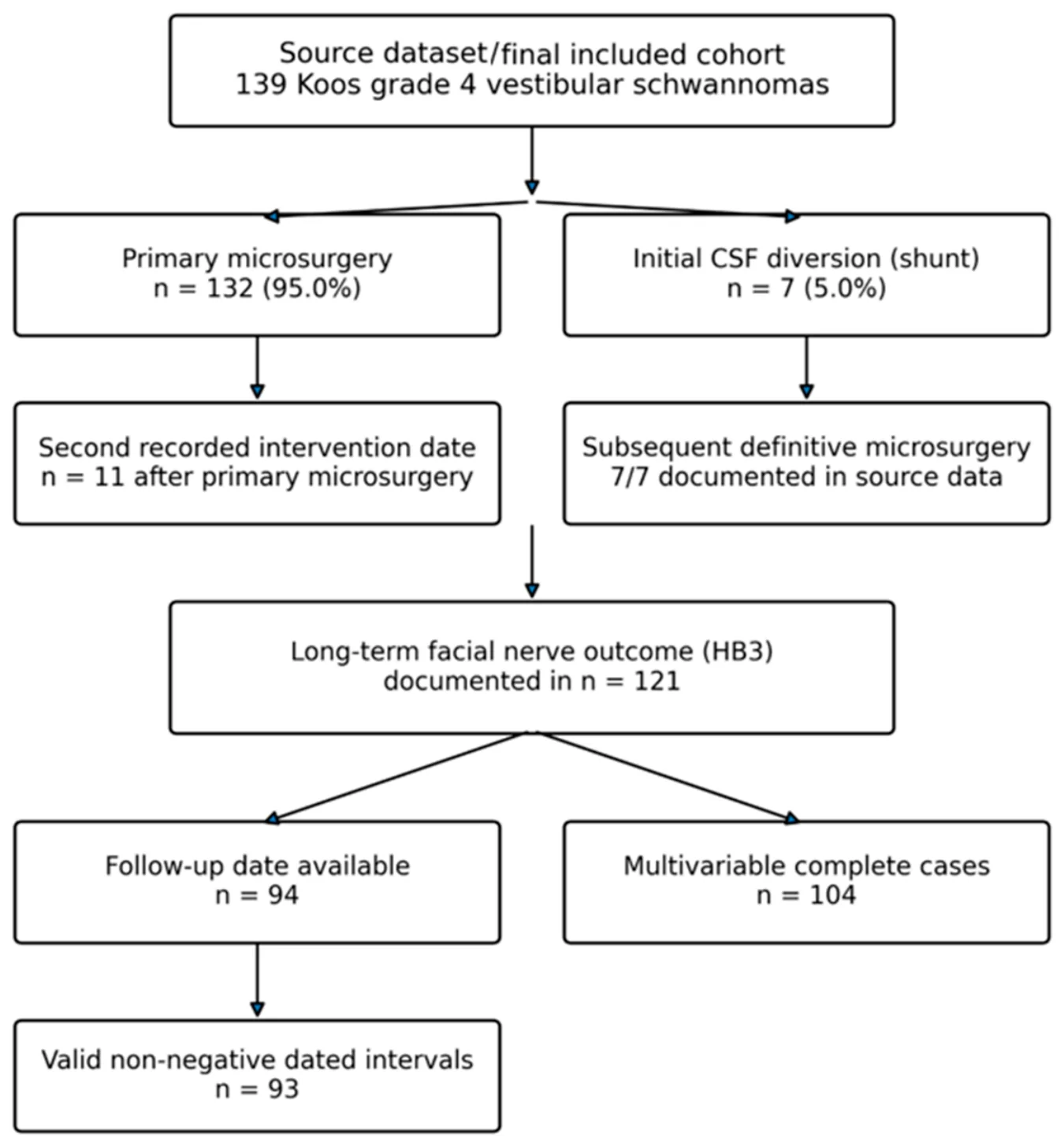
Patient and analysis flow chart. The diagram summarizes the full cohort, initial treatment, subsequent interventions, availability of long-term facial nerve outcome data and dated follow-up, and the multivariable complete-case cohort. CSF, cerebrospinal fluid; HB, House–Brackmann.

Eligible patients were identified through the institutional electronic medical record system. Demographic, clinical, radiological, operative, and follow-up data were retrospectively extracted from admission and discharge records, operative reports, outpatient clinic documentation, audiological examinations, and magnetic resonance imaging (MRI). When follow-up information was incomplete, additional clinical data were obtained from outpatient records or documented follow-up examinations. Only patients with histopathologically confirmed vestibular schwannoma and adequate radiological and clinical follow-up were included in the final analysis. Patients were eligible if they were at least 18 years old at the time of treatment, had a sporadic Koos grade 4 vestibular schwannoma, underwent definitive microsurgical tumor resection at our institution, and had preoperative and early postoperative contrast-enhanced MRI available for review. Patients with neurofibromatosis type 2, biopsy without definitive tumor resection, insufficient clinical or radiological documentation, definitive treatment at another institution, or a histopathological diagnosis other than vestibular schwannoma were excluded (Figure 1).

### 2.2. Tumor Classification

All tumors fulfilled the radiological criteria for Koos grade 4 vestibular schwannoma (brainstem displacement). For the present analysis, they were assigned to a study-specific subdivision adapted from the Hannover T4a/T4b distinction: Koos 4a indicated brainstem compression without fourth ventricular compression, and Koos 4b indicated brainstem displacement with fourth ventricular compression. This terminology was used as a descriptive study variable and is not presented as a validated new classification or as a replacement for the Hannover system. Tumor size was measured on preoperative contrast-enhanced MRI as the maximum extracanalicular diameter in millimeters.

### 2.3. Clinical Symptoms and Functional Outcomes

Clinical symptoms and functional outcomes were assessed preoperatively, immediately after the first microsurgical intervention, and at the last available clinical follow-up. Facial nerve function was categorized by the House–Brackmann (HB) system as favorable (HB I–II) or unfavorable (HB III–VI). Hearing was recorded as normal hearing, hearing loss, or anacusis according to the information available in routine clinical and audiological records. Pure-tone averages and speech discrimination scores were not consistently available; consequently, retrospective assignment to AAO-HNS or Gardner–Robertson classes was not possible, and the reported categories must not be interpreted as serviceable hearing outcomes. Tinnitus and dizziness were recorded as present or absent, gait as stable or unstable, and trigeminal function as normal, hypesthesia, or neuralgia. No standardized vestibular laboratory dataset (caloric testing, video head impulse testing, vestibular-evoked myogenic potentials, or posturography) was available for longitudinal analysis. Dizziness and gait variables therefore represent chart-documented symptoms and bedside function, not a comprehensive assessment of vestibular function. Missing values were excluded pairwise and percentages were based on available observations (Table 1).

### 2.4. Surgical Treatment

Treatment decisions were made within a multidisciplinary skull base team based on patient age, neurological status, hearing function, radiological findings, and overall clinical presentation. Microsurgical resection represented the primary treatment strategy for all tumors included in this study. Patients presenting with symptomatic obstructive hydrocephalus underwent cerebrospinal fluid diversion before definitive tumor resection when clinically indicated (n = 7 patients). The retrosigmoid approach represented the standard surgical corridor and was used in nearly all patients. Continuous intraoperative neurophysiological monitoring of the facial nerve and auditory pathways was routinely employed throughout surgery to facilitate hearing and facial nerve preservation. The primary surgical objective was adequate decompression of the brainstem while preserving neurological function. Gross-total resection was attempted whenever complete tumor removal could be achieved safely. However, if the tumor was densely adherent to the facial nerve, brainstem, or major neurovascular structures, subtotal resection was intentionally accepted to minimize the risk of permanent neurological deficits. This treatment strategy reflects the contemporary concept of functional skull base surgery, in which preservation of neurological function takes precedence over radical tumor removal.

### 2.5. Radiological Assessment

Preoperative and early postoperative contrast-enhanced magnetic resonance images were independently reviewed to evaluate tumor size and the extent of surgical resection. The extent of resection was determined by direct comparison of preoperative and postoperative MRI rather than operative reports. Gross-total resection was defined as complete absence of residual contrast-enhancing tumor on postoperative imaging. Subtotal resection was defined as a small residual contrast-enhancing tumor intentionally left adherent to critical neurovascular structures, whereas partial resection referred to substantial residual enhancing tumor following decompressive surgery.

### 2.6. Outcome Assessment

Neurological outcome was evaluated preoperatively, immediately after the first microsurgical intervention, and at the last documented clinical follow-up. The primary endpoint was facial nerve function at that last available follow-up; the term does not denote a prespecified minimum duration. Follow-up dates were available for 94 patients, and only valid non-negative intervals were summarized. Secondary outcomes included chart-documented hearing category, tinnitus, dizziness, gait instability, trigeminal symptoms, extent of resection, operative duration, hospital stay, complications, and secondary intervention. Facial nerve function was dichotomized as favorable (HB I–II) or unfavorable (HB III–VI). Hearing was categorized as normal, hearing loss, or anacusis; quantitative data required for AAO-HNS or Gardner–Robertson classification were not consistently available. Postoperative complications were extracted from medical records.

### 2.7. Statistical Analysis

Continuous variables are presented as mean +/− standard deviation or median with interquartile range, depending on distribution. Categorical variables are reported as frequencies and valid percentages. Group comparisons used the Mann–Whitney U, Pearson chi-square, or Fisher exact tests as appropriate. Follow-up was recalculated as the interval between first operation and documented follow-up date; negative intervals were excluded. The endpoint was favorable facial nerve function (HB I–II) at the last documented follow-up. Univariable binary logistic regressions were followed by an exploratory multivariable complete-case model including age, sex, study-specific Koos subtype, tumor diameter, gross-total resection, and operative duration. Operative durations of zero, which represented absence of a microsurgical procedure at the first intervention, were treated as unavailable for the operative duration model. We compared patients included in the complete-case model with all excluded patients using the Fisher exact or Mann–Whitney U tests. Variance inflation factors (VIFs) were calculated for all model predictors. To address the anatomical overlap between subtype and diameter, we fitted two sensitivity models, alternately omitting tumor diameter or subtype. The results are reported as odds ratios (ORs) with 95% confidence intervals and two-sided *p*-values; *p* < 0.05 was considered significant. Analyses were performed and independently reproduced in Python 3.12.13 using pandas 2.2.3, NumPy 2.3.5, and SciPy 1.17.0.

## 3. Results

### 3.1. Patient and Tumor Characteristics

**Figure 2:**
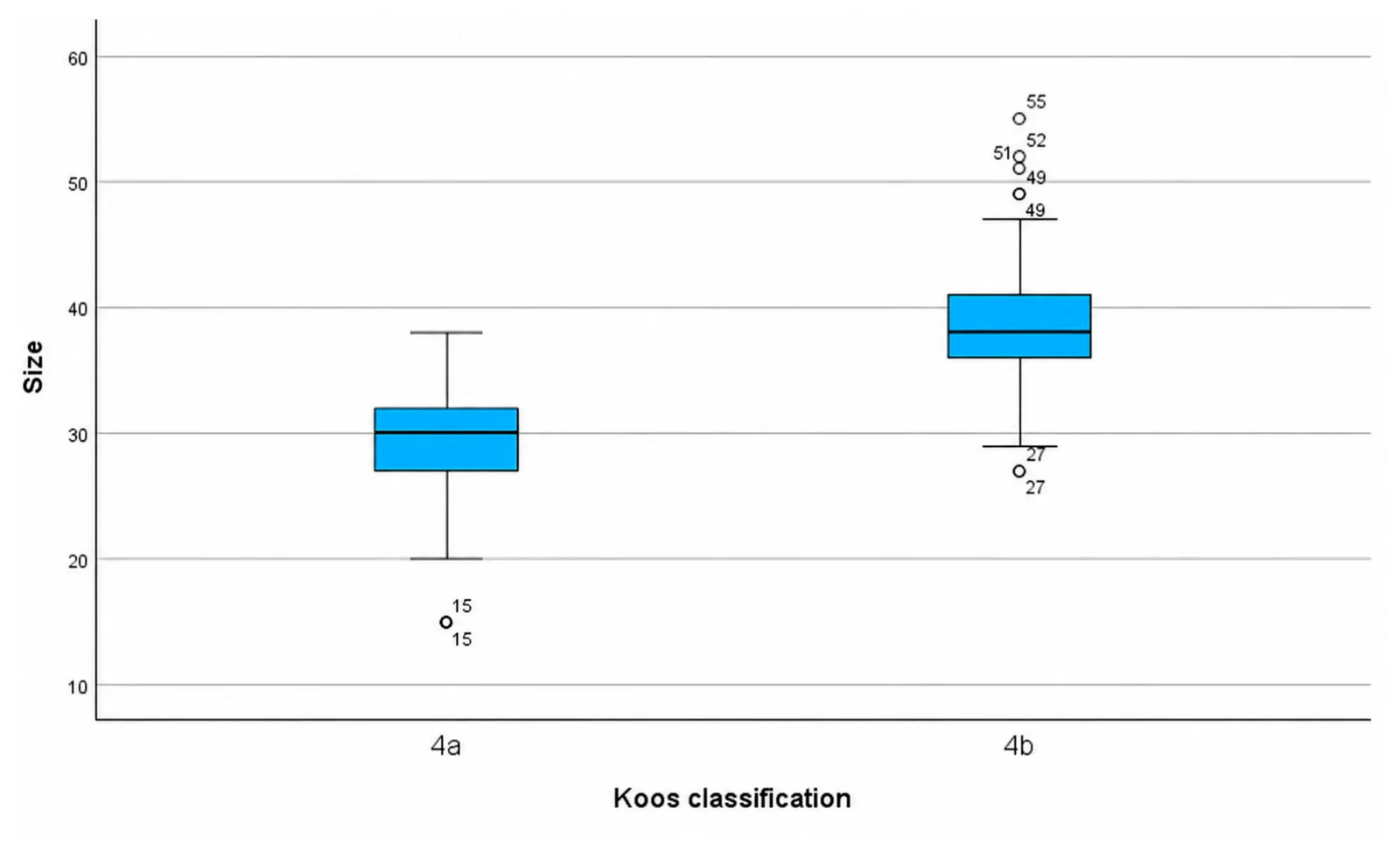
Tumor size according to the study-specific Koos 4a/4b subdivision adapted from the Hannover T4a/T4b distinction. Boxes represent the interquartile range, center lines the median, and plotted points individual observations. Koos 4b tumors were significantly larger than Koos 4a tumors (Mann–Whitney U test, *p* < 0.001).

A total of 139 consecutive patients with unilateral Koos grade 4 vestibular schwannomas met the inclusion criteria and were included in the final analysis. The cohort comprised 76 women (54.7%) and 63 men (45.3%), with a mean age at first intervention of 51.3 ± 13.9 years. Patient age demonstrated an approximately normal distribution, with most patients undergoing surgery between the fourth and seventh decades of life. Among 138 patients with documented tumor laterality, 73 (52.9%) had right-sided tumors and 65 (47.1%) had left-sided tumors. The mean maximum extracanalicular tumor diameter was 33.6 ± 6.1 mm (n = 138), ranging from 15 to 55 mm (Figure 2). Despite all tumors fulfilling the criteria for Koos grade 4 disease, tumor size demonstrated moderate variability, with most lesions measuring between 28 and 40 mm. Using the study-specific anatomical subclassification, 75 tumors (54.0%) were classified as Koos 4a and 64 tumors (46.0%) as Koos 4b, resulting in a nearly balanced distribution between both anatomical subgroups. Tumor size differed significantly between subtypes. Patients with Koos 4a tumors had a median tumor diameter of approximately 30 mm (interquartile range [IQR] 28–32 mm), whereas Koos 4b tumors demonstrated a median diameter of approximately 38 mm (IQR 35–41 mm). This difference was highly significant (Mann–Whitney U = 4347.0; Z = 8.463; *p* < 0.001), confirming that Koos 4b tumors represented the more extensive anatomical subtype.

### 3.2. Surgical Characteristics

Primary microsurgical resection was performed in 132 of 139 patients (95.0%), whereas 7 patients (5.0%) initially underwent cerebrospinal fluid diversion (ventriculoperitoneal shunt placement) because of symptomatic hydrocephalus before definitive tumor surgery. During follow-up, 14 patients required a second intervention. Among these, repeat microsurgical resection was the most common procedure (12 patients; 85.7%), while one patient underwent Gamma Knife radiosurgery (7.1%) and one underwent extra-ventricular drainage (7.1%) in the immediate postoperative period. Of the eight patients initially treated by microsurgery who required further intervention, five (62.5%) underwent repeat microsurgical resection, one (12.5%) received Gamma Knife radiosurgery, and two (25.0%) required CSF diversion. All seven patients initially treated with cerebrospinal fluid shunting subsequently underwent definitive microsurgical tumor resection. The OR duration of primary microsurgical resection was available for 121 patients. Median operative time was 215 min (interquartile range width, 180.5 min; range, 63–580 min), reflecting substantial variability in surgical complexity. Secondary microsurgical procedures were documented in 12 patients, with a median operative time of 140 min (interquartile range width, 186.3 min; range, 89–450 min). The retrosigmoid approach represented the standard operative corridor and was used in 131 of 132 primary microsurgical procedures (99.2%), whereas only one patient (0.8%) underwent a translabyrinthine approach. Among secondary resections, the retrosigmoid approach was employed in 9 of 12 procedures (75.0%), while 3 patients (25.0%) underwent a translabyrinthine approach.

### 3.3. Radiological Extent of Resection

Radiological extent of resection was assessed by comparing preoperative and early postoperative contrast-enhanced MRI. Among the 132 primary microsurgical resections, gross-total resection (GTR) was achieved in 97 patients (73.5%), subtotal resection (STR) in 33 patients (25.0%), and partial resection (PR) in 2 patients (1.5%). Extent of resection following secondary microsurgical procedures was available for all 12 patients: gross-total resection was achieved in 6 patients (50.0%), subtotal resection in 5 patients (41.7%), and partial resection in 1 patient (8.3%). The lower rate of complete tumor removal following repeat surgery most likely reflects the increased technical complexity of revision procedures.

### 3.4. Length of Hospital Stay

Hospital stays following the primary intervention were documented for 136 patients. Median length of stay was 10 days (IQR 8–11 days). Although most patients were discharged within two weeks after surgery, a small number required prolonged hospitalization of up to 40 days, reflecting postoperative complications or complex clinical courses. After secondary interventions, median hospital stay was 11 days (IQR 9–22.5 days), with one patient remaining hospitalized for 61 days.

### 3.5. Postoperative Complications

Overall, 19 of 139 patients (13.7%) experienced at least one postoperative complication, whereas 120 patients (86.3%) had an uncomplicated postoperative course (Table 2). The most common complication was cerebrospinal fluid leakage, occurring in six patients (4.3%), followed by abducens nerve palsy in four patients (2.9%). Wound infection and postoperative hematoma each occurred in two patients (1.4%). Cerebellar edema, glossopharyngeal nerve palsy, hydrocephalus, aspiration pneumonia, and frontal bone impression fracture were each observed in one patient (0.7%). Among the 14 patients undergoing secondary intervention, only two complications were recorded, consisting of one postoperative hematoma and one abducens nerve palsy. The remaining 12 patients (85.7%) experienced no procedure-related complications.

### 3.6. Follow-Up and Regression Analyses

A follow-up date was available for 94 patients. Among 93 valid intervals, median follow-up was 11.6 months (IQR 3.0–40.4; range 0.3–136.9), and mean follow-up was 26.6 +/− 32.8 months. Forty-eight intervals were shorter than 12 months. HB grade at the last available follow-up was documented in 121 patients, including 31 without a corresponding dated visit. Thus, ‘last follow-up’ is the appropriate endpoint label and should not be interpreted as a uniform long-term interval.

**Figure 3:**
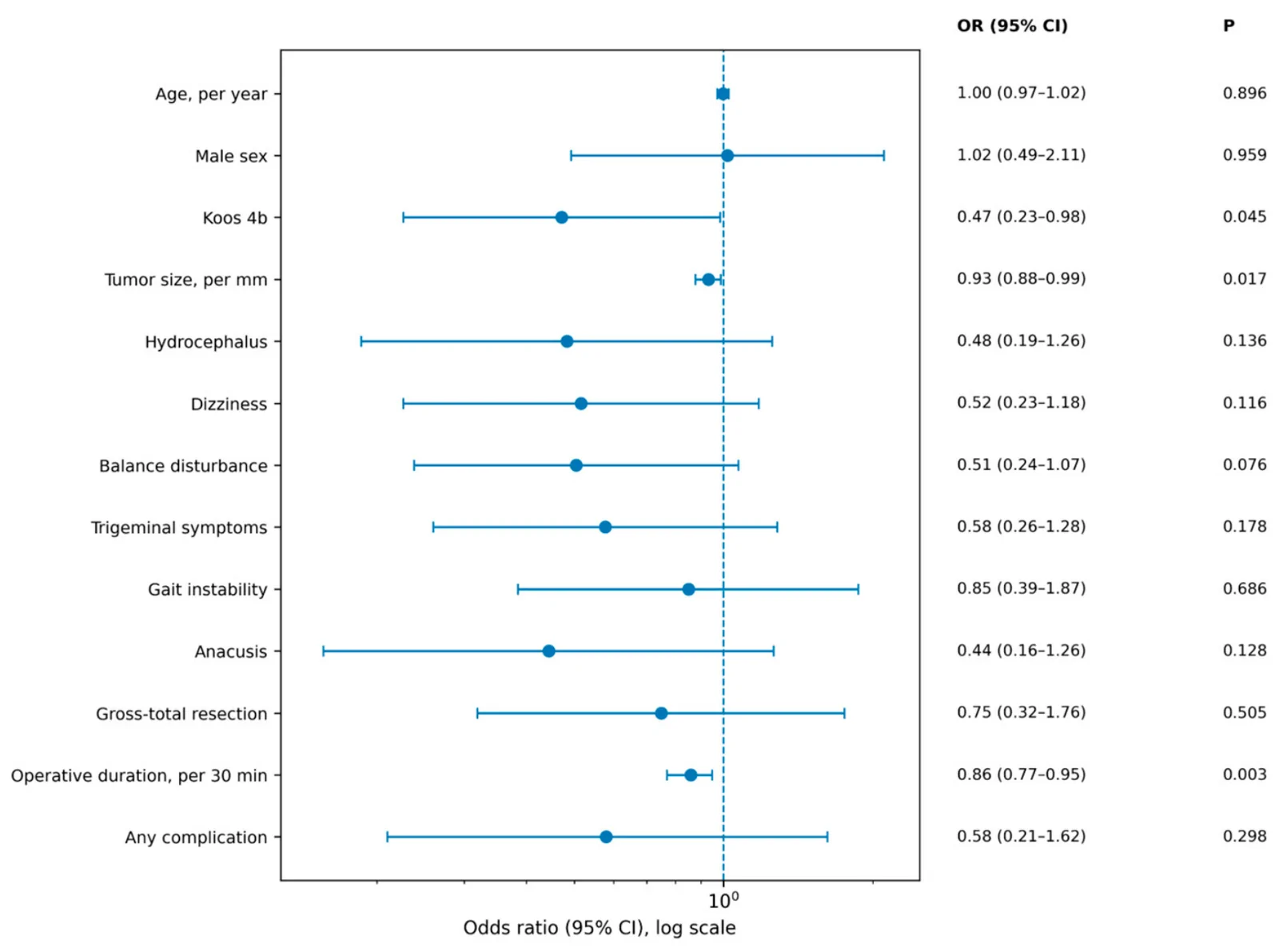
Forest plot of univariable logistic regression analyses for favorable facial nerve outcome at the last documented follow-up (HB I–II). Points indicate ORs and horizontal lines 95% CIs; the vertical line denotes OR = 1. Operative duration is modeled per 30 min increase.

**Figure 4:**
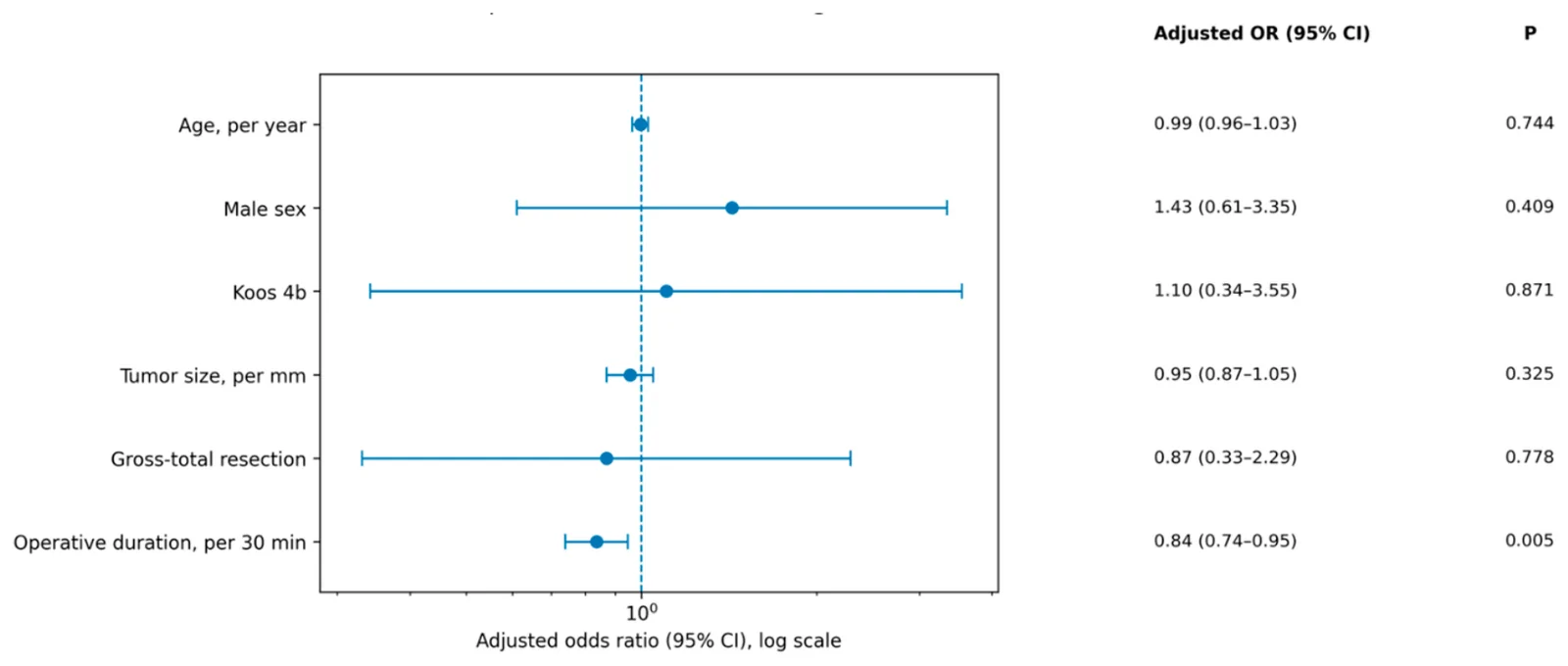
Forest plot of the exploratory multivariable complete-case model for favorable facial nerve outcome at the last documented follow-up (HB I–II; n = 104). Adjusted ORs are shown with 95% CIs. Operative duration is modeled per 30 min increase.

The exploratory univariable analysis showed lower odds of favorable facial nerve outcome for Koos 4b (OR 0.47, 95% CI 0.23–0.98; *p* = 0.045), larger diameter (OR per mm 0.93, 95% CI 0.88–0.99; *p* = 0.017), and longer operative duration (OR per 30 min 0.86, 95% CI 0.77–0.95; *p* = 0.003) (Figure 3, Table 3). The complete-case model included 104 patients, with 61 favorable and 43 unfavorable outcomes. Operative duration remained associated with lower odds of favorable outcome (adjusted OR per 30 min 0.84, 95% CI 0.74–0.95; *p* = 0.005), whereas the study-specific subtype estimate was imprecise (adjusted OR 1.10, 95% CI 0.34–3.55; *p* = 0.871) (Figure 4, Table 4). VIFs ranged from 1.03 to 1.97 (subtype 1.97; diameter 1.86), providing no evidence of problematic statistical multicollinearity. Sensitivity analyses were consistent: after omission of diameter, the adjusted OR for Koos 4b was 0.76 (95% CI 0.31–1.87; *p* = 0.545), and after omission of subtype, the adjusted OR per millimeter for diameter was 0.96 (95% CI 0.89–1.03; *p* = 0.253). Operative duration remained associated in both models (adjusted OR per 30 min 0.83, *p* = 0.004; and 0.84, *p* = 0.004), (Table 5).

## 4. Discussion

This study describes surgical and functional outcomes in 139 consecutive patients with Koos grade 4 vestibular schwannomas and explores a descriptive subdivision adapted from the established Hannover T4a/T4b distinction [7]. Its purpose was not to introduce a competing classification, but to test whether fourth ventricular involvement added prognostic information within Koos grade 4 disease. The subgroup with fourth ventricular compression had larger tumors and greater preoperative neurological burden. Its association with facial nerve outcome in univariable analysis was not retained in a mutually adjusted exploratory model; however, subtype and diameter capture overlapping anatomy, and the wide confidence interval does not support a definitive claim of no independent effect. Operative duration showed the most robust association in the fitted model, but is best viewed as a downstream marker of surgical difficulty, adherence, and anatomical complexity rather than a modifiable causal exposure.

Large vestibular schwannomas remain among the most technically demanding lesions in skull base surgery because tumor removal must be balanced against preservation of neurological function [2,5,10,11,12]. Historically, gross-total resection was considered the principal surgical objective; however, increasing emphasis on postoperative facial nerve function and quality of life has shifted contemporary practice toward maximal safe resection, accepting a small residual when further dissection would place the facial nerve or brainstem at unacceptable risk [5,8,13,14]. Current EANO, CNS, and EANS guidance supports individualized treatment strategies that balance tumor control and functional preservation [3,4,5].

In the present cohort, gross-total resection was achieved in 73.5% of patients, whereas 25.0% underwent subtotal resection and 1.5% partial resection. Radiological extent of resection was determined by direct comparison of preoperative and early postoperative contrast-enhanced MRI rather than operative reports. The observed gross-total resection rate is within the range reported in contemporary series of large vestibular schwannomas [8,10,12]. Schneider et al. reported gross-total resection in approximately three-quarters of patients and observed favorable facial nerve outcomes after near-total resection [8], while Sughrue et al. questioned whether more extensive resection necessarily improves long-term tumor control [13]. These findings, together with systematic evidence in large tumors, support a function-preserving approach when complete removal would substantially increase neurological risk [5,14].

The overall postoperative complication rate of 13.7% observed in our study is consistent with the morbidity reported in contemporary series and systematic analyses of large vestibular schwannoma surgery [5,10,11,12]. Cerebrospinal fluid leakage was the most frequent complication (4.3%), followed by abducens nerve palsy (2.9%). A meta-analysis of large tumors treated through a retrosigmoid approach reported cerebrospinal fluid leakage in 7.8% [11], while large contemporary cohorts and consensus reviews likewise identify CSF-related complications and cranial nerve deficits among the principal postoperative morbidities [5,10,12].

Preservation of facial nerve function remains a major determinant of functional outcome after vestibular schwannoma surgery [10,14,15]. Immediate postoperative deterioration followed by subsequent recovery has been described in contemporary surgical series [10,12,15]. In our cohort, favorable House–Brackmann grades (I–II) decreased from 98.5% preoperatively to 36.0% immediately after surgery and improved to 58.7% at last follow-up. Published data consistently show that facial nerve outcome worsens with increasing tumor size, while function may improve during follow-up after the early postoperative period [10,11,12,14,15].

Hearing preservation is particularly challenging in large vestibular schwannomas [11,12,16]. Most patients already had chart-documented hearing loss before surgery, and anacusis was more frequent postoperatively. These descriptive categories do not distinguish serviceable from non-serviceable hearing. Because pure-tone averages and speech discrimination scores were not consistently retrievable, AAO-HNS or Gardner–Robertson classes could not be assigned retrospectively [17]. The hearing findings should therefore be interpreted as broad clinical documentation rather than quantitative audiological outcomes. Brainstem decompression and facial nerve preservation were prioritized in this advanced-tumor cohort, consistent with contemporary recommendations [3,4,5].

Fourth ventricular involvement parallels the Hannover T4a/T4b distinction and appears to mark greater anatomical and clinical severity [7]. Harati et al. reported greater neurological burden in T4b tumors, including ataxia and hydrocephalus, while other large-tumor cohorts examined related surgical outcomes [7,8,9]. Our unadjusted findings are directionally consistent with this research. However, the study-specific terminology is unvalidated, the subtype is closely related to diameter, and mutually adjusted estimates were imprecise. Accordingly, the present study neither establishes a new classification nor proves that fourth ventricular involvement lacks prognostic value; it provides a hypothesis-generating description that requires external validation.

The present study has several strengths. It represents one of the larger contemporary single-center cohorts exclusively investigating surgically treated Koos grade 4 vestibular schwannomas. All patients were treated at a high-volume skull base center using a standardized microsurgical strategy and routine intraoperative neurophysiological monitoring. Furthermore, extent of resection was determined by postoperative MRI rather than surgeon assessment, increasing methodological robustness and reducing observer bias. Neurological outcomes were assessed at three clinically relevant time points, allowing evaluation of both immediate postoperative morbidity and long-term functional recovery. In addition, regression analyses enabled assessment of whether the observed association between anatomical subtype and facial nerve outcome persisted after accounting for relevant clinical and surgical factors.

Several limitations warrant emphasis. The retrospective single-center design permits selection and documentation bias. Endpoint availability differed: HB grade at the last follow-up was documented for 121/139 patients, a valid dated interval for 93, and all model variables for 104. Compared with the 35 patients excluded from the complete-case model, included patients had less preoperative hydrocephalus (12.9% vs. 31.4%; *p* = 0.020) and longer recorded operative duration (median 215 vs. 152 min; *p* = 0.002), indicating that complete-case selection was not entirely random. Audiological data lacked consistent pure-tone averages and speech discrimination scores, preventing AAO-HNS or Gardner–Robertson classification. Dizziness and gait were chart-derived; standardized caloric testing, video head impulse testing, vestibular-evoked myogenic potentials, posturography, and a validated dizziness questionnaire were not available longitudinally. These variables cannot represent comprehensive vestibular outcomes. Central compensation after unilateral vestibular loss commonly evolves over weeks to months and depends on central, visual, proprioceptive, and rehabilitation-related factors; it may explain part of the improvement in dizziness and gait, but cannot be quantified from our data [18,19]. Postoperative tinnitus and trigeminal symptoms were documented much less consistently than facial function, particularly immediately after surgery, because they were not part of a standardized prospective assessment; their percentages are susceptible to documentation bias. The subdivision is study-specific and requires validation. Although all VIFs were below 2 and sensitivity models were consistent, subtype and diameter remain conceptually overlapping measures of tumor extent, and confidence intervals were wide. The model contained six predictors and only 43 unfavorable events (approximately 7.2 events per predictor), below the traditional 10-events-per-variable heuristic; overfitting and imprecision remain possible. No a priori power calculation was performed, so the analysis is hypothesis-generating. Finally, operative duration is a post-exposure marker likely prolonged by adherence and dissection difficulty. Reverse causation and residual confounding preclude interpreting duration as causal or actionable. Follow-up was variable and sometimes undated; the endpoint is therefore the last documented follow-up rather than a standardized long-term outcome.

## 5. Conclusions

This study-specific subdivision, adapted from Hannover T4a/T4b anatomy, describes differences in tumor extent and preoperative burden within Koos grade 4 disease. Its independent prognostic value was not established, and the current data are insufficient to recommend it as a stand-alone prognostic tool. Operative duration was associated with facial nerve outcome in an exploratory model but should be regarded as a marker of surgical complexity, not a causal treatment target. External validation with standardized audiovestibular testing, predefined follow-up, and adequately powered modeling is needed.

## Figures and Tables

**Table 1:** Symptoms and functional outcomes at the three assessment timepoints. Values are presented as n/N (%), where N is the number of patients with available data for the respective variable and timepoint. Missing observations were excluded pairwise. Favorable facial nerve function was defined as House–Brackmann grades I–II and unfavorable function as grades III–VI. HB, House–Brackmann.

Symptom	Expression	Preoperative	Immediately Postoperative	Last Follow-Up
Facial nerve function	Favorable (HB I–II)	132/134 (98.5%)	49/136 (36.0%)	71/121 (58.7%)
	Unfavorable (HB III–VI)	2/134 (1.5%)	87/136 (64.0%)	50/121 (41.3%)
Hearing	Normal	5/138 (3.6%)	2/102 (2.0%)	1/117 (0.9%)
	Hearing loss	115/138 (83.3%)	37/102 (36.3%)	49/117 (41.9%)
	Anacusis	18/138 (13.0%)	63/102 (61.8%)	67/117 (57.3%)
Tinnitus	Yes	77/130 (59.2%)	13/28 (46.4%)	22/54 (40.7%)
	No	53/130 (40.8%)	15/28 (53.6%)	32/54 (59.3%)
Dizziness	Yes	81/125 (64.8%)	95/113 (84.1%)	31/87 (35.6%)
	No	44/125 (35.2%)	18/113 (15.9%)	56/87 (64.4%)
Gait stability	Stable	84/128 (65.6%)	34/108 (31.5%)	75/97 (77.3%)
	Unstable	44/128 (34.4%)	74/108 (68.5%)	22/97 (22.7%)
Trigeminal symptoms	Normal	83/126 (65.9%)	40/58 (69.0%)	48/64 (75.0%)
	Hypoesthesia	37/126 (29.4%)	17/58 (29.3%)	16/64 (25.0%)
	Neuralgia	6/126 (4.8%)	1/58 (1.7%)	0/64 (0.0%)

**Table 2:** Complications after the first intervention in the full cohort (n = 139). Values are presented as number of patients and percentage of the total cohort. CSF, cerebrospinal fluid.

Complications	n	%
No complication	120	86.3
CSF leak	6	4.3
Abducens palsy	4	2.9
Wound infection	2	1.4
Hematoma	2	1.4
Cerebellar edema	1	0.7
Glossopharyngeal palsy	1	0.7
Hydrocephalus	1	0.7
Aspiration pneumonia	1	0.7
Impression fracture of the frontal bone	1	0.7
Total	139	100.0

**Table 3:** Univariable logistic regression analyses for favorable facial nerve outcome at the last documented follow-up (HB I–II). ORs below 1 indicate lower odds of a favorable outcome. Age and tumor size are modeled per one-year and one-millimeter increase; operative duration per 30 min increase. CI, confidence interval; OR, odds ratio.

Predictor	OR	95% CI	*p*
Age, per year	1.00	0.97–1.02	0.896
Male sex	1.02	0.49–2.11	0.959
Koos 4b	0.47	0.23–0.98	0.045
Tumor size, per mm	0.93	0.88–0.99	0.017
Hydrocephalus	0.48	0.19–1.26	0.136
Dizziness	0.52	0.23–1.18	0.116
Balance disturbance	0.51	0.24–1.07	0.076
Trigeminal symptoms	0.58	0.26–1.28	0.178
Gait instability	0.85	0.39–1.87	0.686
Anacusis	0.44	0.16–1.26	0.128
Gross-total resection	0.75	0.32–1.76	0.506
Operative duration, per 30 min	0.86	0.77–0.95	0.003
Any complication	0.58	0.21–1.62	0.298

**Table 4:** Exploratory multivariable logistic regression for favorable facial nerve outcome at the last documented follow-up (HB I–II; complete-case n = 104). All listed predictors were entered simultaneously. Because study-specific subtype and tumor diameter represent related measures of tumor extent, coefficients require cautious interpretation; CI, confidence interval.

Predictor	Adjusted OR	95% CI	*p*
Age, per year	0.99	0.96–1.03	0.744
Male sex	1.43	0.61–3.35	0.409
Koos 4b	1.10	0.34–3.55	0.871
Tumor size, per mm	0.95	0.87–1.05	0.325
Gross-total resection	0.87	0.33–2.29	0.778
Operative duration, per 30 min	0.84	0.74–0.95	0.005

**Table 5:** Comparison of patients included in the multivariable complete-case model and those excluded from this model. Values are median [IQR] or n/N (%). *p*-values are from Mann–Whitney U or Fisher exact tests. Outcome availability among excluded patients was limited to 17/35.

Variable	Included (n = 104)	Excluded (n = 35)	*p*-Value
Age, years	50.5 [40.0–60.0]	52.0 [41.0–62.5]	0.495
Male sex	52/104 (50.0%)	11/35 (31.4%)	0.077
Koos 4b	47/104 (45.2%)	17/35 (48.6%)	0.845
Tumor diameter, mm	32.0 [29.0–37.0]	33.5 [28.2–39.0] (n = 34)	0.569
Preoperative hydrocephalus	13/101 (12.9%)	11/35 (31.4%)	0.020
Gross-total resection	75/104 (72.1%)	22/28 (78.6%)	0.632
Operative duration, min	215 [140.8–330.0]	152 [102.5–218.0] (n = 24)	0.002
Favorable HB at last follow-up	61/104 (58.7%)	10/17 (58.8%)	1.000

## Data Availability

The patient-level data generated and/or analyzed during the present study are not publicly available due to patient privacy, ethical, and institutional data protection requirements. Access to the de-identified data may be granted by the corresponding author upon reasonable request, subject to approval by the responsible institutional review board/data protection authority and the execution of an appropriate data use agreement.

## Abbreviations

The following abbreviations are used in this manuscript:

aOR/aORs	Adjusted odds ratio(s)
C3N-MH	Comprehensive Center of Clinical Neurosciences and Mental Health
CI/CIs	Confidence interval(s)
CNS	Congress of Neurological Surgeons
CSF	Cerebrospinal fluid
EANO	European Association of Neuro-Oncology
EANS	European Association of Neurosurgical Societies
GTR	Gross-total resection
HB	House–Brackmann
IQR	Interquartile range
MRI	Magnetic resonance imaging
OR/ORs	Odds ratio(s)
PR	Partial resection
STR	Subtotal resection
VP	Ventriculoperitoneal
VS	Vestibular schwannoma(s)
n	Number of observations or patients
N	Number of patients with available data
*p*	*p*-value
U	Mann–Whitney U statistic
Z	Standardized test statistic
χ^2^	Chi-square statistic/test
